# Pollen–stigma interactions in Brassicaceae: complex communication events regulating pollen hydration

**DOI:** 10.1093/jxb/eraa117

**Published:** 2020-05-09

**Authors:** Maurice Bosch, Ludi Wang

**Affiliations:** Institute of Biological, Environmental and Rural Sciences (IBERS), Aberystwyth University, Plas Gogerddan, Aberystwyth, UK

**Keywords:** Actin cytoskeleton, Brassicaceae, cellular communication, hydration, pollen recognition, pollen–stigma interaction, self-incompatibility

## Abstract

This article comments on:

**Rozier F, Riglet L, Kodera C, Bayle V, Durand E, Schnabel J, Gaude T, Fobis-Loisy I**. 2020. Live-cell imaging of early events following pollen perception in self-incompatible *Arabidopsis thaliana*. Journal of Experimental Botany 71, 2513–2526.

**The process of plant reproduction is responsible for much of our food supply, with fertility and seed set critical for crop yield and thus food security. The first stage of male–female recognition in flowering plants takes place when pollen lands on the surface of the stigma. In Brassicaceae, compatible pollen will hydrate and germinate, leading to successful fertilization, while these processes are severely impaired in incompatible pollen, preventing fertilization. Rozier *et al.* (2020) have developed a semi-*in vivo* live-cell imaging system to investigate early pollen–stigma interactions in both compatible and incompatible pollinations in *Arabidopsis thaliana*.**

Successful fertilization in flowering plants involves multiple communication events between the pollen and the pistil (Dresselhaus and Franklin-Tong, 2013). In Brassicaceae, this communication starts soon after pollen is captured on stigmatic papilla cells. A captured desiccated pollen grain has to hydrate first to become metabolically active before it can germinate and develop a pollen tube that penetrates the stigmatic cell wall and grows through the apoplastic space down to the ovary for fertilization (Kandasamy *et al.*, 1994). The dry surface of stigmas in the Brassicaceae represents an important checkpoint for selective pollen hydration (Dickinson, 1995). A compatible pollination triggers cellular responses in the stigma to transfer water to the desiccated pollen while in the Brassicaceae species that exhibit the sporophytic self-incompatibility (SI) system, pollen hydration is significantly inhibited following an incompatible pollination (Dickinson, 1995). Even when incompatible pollen manages to by-pass the first checkpoint, for instance under conditions of high humidity, these tubes are arrested at the point of stigmatic cell wall penetration (Zuberi and Dickinson, 1985).

Although we have good knowledge of the SI-induced signalling events leading to self-pollen rejection in the Brassicaceae (briefly discussed later; see also Box 1), studies on the cellular and molecular responses of stigmatic papilla cells following both compatible and self-incompatible pollination are challenging, with many aspects remaining unclear.

**Box 1. Key components involved in early pollen–stigma communication events in Brassicaceae**

Following pollen deposition, two distinct signalling systems are thought to regulate pollen acceptance or rejection: the ‘basal’ compatible pollen response pathway (A) and the self-incompatible pathway (B).

Signalling factors in the stigma that trigger the compatible pathway remain largely unknown (A). PCP-B pollen coat proteins could act as ligands for triggering the compatibility pathway that initiates water transfer from stigmatic papilla cells. EXO70A1 regulates polarized secretion of vesicles (in Arabidopsis) or MVBs (in *Brassica*) in the papilla cells, which is essential for the hydration of pollen grains (A).

The self-incompatibility pathway leading to self-pollen rejection is initiated by the interaction of the stigma-expressed SRK receptor with the cognate pollen *S*-locus CRP SCR/SP11 (B). This interaction results in the phosphorylation of SRK, which in turn phosphorylates two other proteins: M-locus protein kinase (MLPK) and Armadillo repeat containing 1 (ARC1) with E3 ubiquitin ligase activity. The activated ARC1 facilitates the proteasomal degradation of the exocyst subunit EXO70A1. EXO70A1 therefore acts at the interconnection of the two signalling pathways, while it appears that these two pathways are separated upstream of EXO70A1. The functional role of the increase in cytosolic Ca^2+^ observed after a compatible pollination (A), and even more dramatic after an incompatible pollination (B) remains to be resolved. Note that the ACA13-mediated export of Ca^2+^ from the papilla cells to the pollen, only induced by compatible pollination, is not shown here.

Reorganization and focalization of the actin cytoskeleton (shown as orange dashed lines) have been observed in the stigma at the contact site with compatible pollen and the pollen tube (A, C). It remains to be confirmed if this actin reorganization is triggered by the mechanical pressure produced by pollen tubes at the site of cell wall penetration and/or if it is a direct consequence of a signalling cascade (e.g. involving Ca^2+^ and other factors altering the activity of ABPs) initiated by the compatibility response.

The observation that, facilitated by high humidity, some hydrated incompatible pollen grains can germinate but not penetrate the papilla cell wall (C) suggests that this penetration step may represent an additional checkpoint (besides the pollen hydration step) to help ensure that incompatible pollen cannot achieve fertilization. This penetration step may require the regulated secretion of enzymes along the pollen–papilla contact sites to facilitate pollen tube growth through the cell wall matrix (C).

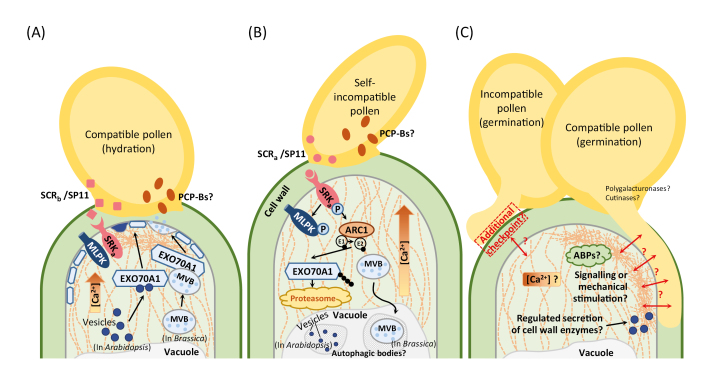

Several studies have used imaging techniques to investigate changes in the stigmatic papilla cells during self- and cross-pollination (e.g. Dickinson, 1995; Iwano *et al.*, 2007; Hiroi *et al.*, 2013; Safavian and Goring, 2013). The new system described by Rozier *et al.* (2020), utilizing engineered Arabidopsis SI lines in combination with an elegant imaging set-up, allows live-cell imaging of morphological and physiological changes in Arabidopsis following compatible and self-incompatible pollinations.

## What controls pollen recognition and hydration?

Following capture of pollen grains on the stigmatic surface, pollen coat material mainly composed of proteins and lipids forms a ‘foot’ that establishes continuity at the pollen–papilla cell interface (Kandasamy *et al.*, 1994). Rozier *et al.* (2020) carried out semi-*in vivo* assays using live-cell imaging to record early pollination events. Using the ratio of the long and wide pollen axis (L/W) as a proxy for pollen hydration, they showed that compatible pollen need to pass below a threshold value of L/W=1.4 before they start germinating. As shown before in *Brassica* (Dickinson, 1995; Hiroi *et al.*, 2013), the hydration of compatible pollen starts within a few minutes after pollen deposition. The kinetics of compatible pollen hydration turned out to be biphasic—a rapid hydration phase during the first 10 min is followed by a phase where there is little further hydration and pollen germination occurs (Rozier *et al.*, 2020). Most incompatible pollen showed little sign of hydration (Rozier *et al.*, 2020). As suggested by Dickinson (1995), this indicates that the SI response operates within the first few minutes after deposition of incompatible pollen and that one of the main consequences is blocking of pollen hydration.

Secreted cysteine-rich proteins (CRPs) found in the pollen coat have been shown to be involved in cellular recognition/communication during the earliest stages of pollen–stigma interaction (reviewed by Marshall *et al.*, 2011). The *S*-locus cysteine-rich/*S*-locus protein 11 (SCR/SP11) is a pollen coat-derived CRP involved in controlling recognition of incompatible pollen. SCR/SP11 acts as the ligand in Brassicaceae SI, binding to the allelic *S*-locus receptor kinase (SRK) in the stigmatic papillae to activate a signalling cascade that inhibits hydration and germination of incompatible pollen (Schopfer *et al.*, 1999; Takayama *et al.*, 2000). Another class of CRPs, called PCP-Bs (pollen coat protein B class), have been identified as key regulators of compatible pollen hydration (Wang *et al.*, 2017). Knockouts of PCP-B-encoding genes exhibited severely impaired pollen hydration. To obtain a better understanding of pollen–stigma communication that establishes compatibility, it will be important to identify the stigma receptor of the PCP-B proteins. Such a ‘compatible’ receptor–ligand module may be responsible for triggering a signalling cascade in the papilla cells that leads to polarized secretion at the pollen contact site.

One of the early cellular regulators of pollen hydration and germination is vesicle trafficking in the papilla cells (Box 1; reviewed in Goring, 2017). Studies in *Brassica oleracea* showed vesicle-like structures in the papilla cell wall following compatible pollination (Dickinson, 1995; Elleman and Dickinson, 1996). More recent studies illustrated that multivesicular bodies (MVBs) in papillae fuse to the plasma membrane beneath the contact point with compatible pollen (Safavian and Goring, 2013). In response to incompatible pollen, MVBs as well as autophagic bodies were found in the vacuole, which may indicate disruption of vesicle trafficking and secretory activity (Safavian and Goring, 2013; Indriolo *et al.*, 2014).

Exocyst-mediated polarized secretion in stigmatic papilla cells was shown to be important for compatible pollen hydration (Samuel *et al.*, 2009; Safavian *et al.*, 2015). EXO70A1, a subunit of the exocyst complex acting as a tethering mediator of polarized secretion, has been shown to be essential for the acceptance of compatible pollen and to be ubiquitinated by the ARM repeat-containing protein ARC1 E3 ligase in the SI pathway (Samuel *et al.*, 2009; Indriolo *et al.*, 2012, 2014; Box 1). Based on L/W ratios reported by Rozier *et al.* (2020), the pollen volume between 2 min and 10 min after compatible pollen deposition increases with ~1500 μm^3^ (equivalent to a 44% increase). This further illustrates the extent of vesicle trafficking and targeted secretion required at the papilla–pollen contact site to accommodate the rapid pollen hydration process in Brassicaceae.

## A function of actin focalization in hydration and germination of compatible pollen?

Remodelling of the actin cytoskeleton, leading to actin focalization at the contact site with compatible pollen, appears to be a hallmark feature in compatible pollen–stigma interactions in Arabidopsis and *Brassica* (Iwano *et al.*, 2007; Rosier *et al.*, 2020). Rosier *et al.* (2020) hypothesize that actin remodelling in stigmatic papillae is triggered by the mechanical pressure produced by the pollen tube at the site of cell wall penetration. Indeed, interaction with microbes and application of physical stimuli have both been shown to cause aggregation of actin microfilaments beneath the contact point in leaf epidermal cells or cotyledons (Jayaraman *et al.*, 2014). How local mechanical stimulation is translated to localized actin remodelling remains to be determined. As an alternative to the mechanical pressure hypothesis, the observed actin focalization may be a result of a signalling cascade initiated by a compatible pollen–stigma recognition event. Calcium plays an important role in controlling actin dynamics (Hepler, 2016) and may, perhaps by altering the properties of specific actin-binding proteins (ABPs), be involved in actin remodelling leading to focalization. Compatible pollinations induce increases in papilla cell Ca^2+^, just below the contact site, with the highest increase observed when the pollen tube penetrates the papilla cell wall (Iwano *et al.*, 2004). Interestingly, a subsequent study showed that compatible pollination triggers the export of Ca^2+^ from papilla cells to germinating pollen, a process promoting successful fertilization and mediated by ACA13, a calmodulin-activated calcium pump, accumulating at the plasma membrane near the pollen attachment site (Iwano *et al.*, 2014).

The function of actin focalization remains to be determined. The fact that the actin cytoskeleton is important for the delivery of secretory vesicles and knowledge that there is polarized delivery of secretory vesicles in the papilla cells to the pollen contact site to promote localized exocyst tethering and membrane fusion (Samuel *et al.*, 2009) suggests the possible involvement of actin focalization in these processes. They may also be involved in changes to the vacuolar network in the apical region of the stigmatic papilla, with the large central vacuole orienting towards the contact site with compatible pollen, perhaps to promote pollen hydration (Iwano *et al.*, 2007). In contrast, the application of self-incompatible pollen grains or pollen coat was associated with a decrease in actin filaments in the apical region and a more disorganized appearance of the vacuolar network in the stigmatic papillae (Iwano *et al.*, 2007). While compatible pollinations clearly stimulate changes in the organization of the actin cytoskeleton and the vacuole as well as altered Ca^2+^ levels and polarized secretion in papilla cells, the level of integration and crosstalk between these events remains largely unknown (see Box 1).

## Penetrating the cell wall barrier

Early electron microscopy studies have shown that, prior to pollen tube penetration, a compatible pollen–stigma interaction triggers an expansion of the outer papilla cell wall layer at the site of interaction (Dickinson, 1995). Following penetration of the papilla cell cuticle and cell wall, the pollen tube grows toward the base of this cell through the cell wall matrix along the surface of the plasma membrane. Papilla cell wall expansion and/or loosening, pollen tube penetration, and subsequent growth through the cell wall matrix clearly require major enzymatic modifications of the papilla cell wall. Yet, besides a functional role in pollen tube penetration implicated for pollen polygalacturonases and cutinases (Hiscock *et al.*, 2002), there are surprisingly few data on cell wall-related enzymes involved in these early pollen–stigma interaction steps. As previously mentioned, a compatible interaction triggers polarized exocytosis in the stigmatic papillae. It will be important to identify the content of these secretory vesicles that are delivered to the pollen–stigma interaction site as these may contain molecules involved in pollen hydration and penetration. In agreement with earlier studies (e.g. Zuberi and Dickinson, 1985), Rozier *et al.* (2020) showed that even when an incompatible pollen manages to germinate and grow a tube, for instance under high humidity conditions, these invariably fail to penetrate the papilla cell wall. This suggests that stigma-derived enzymes required for pollen tube penetration have not been delivered to the stigmatic surface, implicating the existence of an additional checkpoint that prevents the penetration of germinated incompatible pollen when the earlier hydration checkpoint has been breached.

Two interacting signalling pathways have been suggested to operate in the stigma of Brassicaceae that exhibit SI: the ‘basal compatible pollen response pathway’ regulating the acceptance of compatible pollen, and the extensively studied self-incompatible pathway leading to self-pollen rejection (Doucet *et al.*, 2016; Box 1). Future work, utilizing proteomic and transcriptome profiling approaches, will undoubtedly reveal further components playing a part in the early pollen–stigma recognition events involving these two pathways. The availability of Arabidopsis plants harbouring the *Brassica* SI system, along with other genetic tools that can be combined with live-cell imaging approaches utilizing the set-up described by Rozier *et al.* (2020), provides exciting opportunities to further dissect the molecular and physiological mechanisms involved in the early pollen–stigma communication events.
